# Longitudinal CSF and Serum Biomarker Dynamics in Tofersen-Treated *SOD1*-ALS: A Real-World Multicentre Cohort Study

**DOI:** 10.3390/ijms27104208

**Published:** 2026-05-09

**Authors:** Andrea Giordano, Jessica Mandrioli, Federica Cerri, Christian Lunetta, Hamidreza Saebfar, Marcella Catania, Claudia Battipaglia, Laura Leone, Francesca Trojsi, Maria Vizziello, Francesca Gerardi, Matteo Farè, Aida Zulueta, Rachele Piras, Matteo Giacchino, Giulia Gianferrari, Eleonora Dalla Bella, Teuta Domi, Dario Bonanomi, Giuseppe Ganci, Raffaella Lombardi, Giuseppe Lauria, Nilo Riva

**Affiliations:** 1 Neurology 3—Neuroalgology Unit, Fondazione IRCCS Istituto Neurologico Carlo Besta, Via Celoria 11, 20133 Milan, Italy; 2 Department of Medical Biotechnology and Translational Medicine, Università degli Studi di Milano, Via Vanvitelli 32, 20133 Milan, Italy; 3 Department of Neurosciences, Ospedale Civile Baggiovara, Azienda Ospedaliero Universitaria di Modena, Via Giardini 1355, 41126 Modena, Italy; 4 Department of Biomedical, Metabolic and Neural Sciences, University of Modena and Reggio Emilia, Via Campi 287, 41125 Modena, Italy; 5 NEuroMuscular Omnicentre, Fondazione Serena ETS, Piazza dell’Ospedale Maggiore, 3, 20162 Milan, Italy; 6 Neurorehabilitation Department, Istituti Clinici Scientifici Maugeri IRCCS, Via Camaldoli 64, 20138 Milan, Italy; 7 Neurology 8—Dementias and Degenerative Diseases of Central Nervous System Unit, Fondazione IRCCS Istituto Neurologico Carlo Besta, Via Celoria 11, 20133 Milan, Italy; 8 Neurology Unit, First Division of Neurology and Neurophysiopathology, AOU University of Campania “Luigi Vanvitelli”, Piazza Miraglia 2, 80138 Naples, Italy; 9 Division of Neuroscience, IRCCS San Raffaele Scientific Institute, Via Olgettina 60, 20132 Milan, Italy; 10 Neurointerventional Radiology Unit, Fondazione IRCCS Istituto Neurologico Carlo Besta, Via Celoria 11, 20133 Milan, Italy

**Keywords:** *SOD1*-ALS, tofersen, neurofilament light chain, GFAP, UCHL–1, total Tau, cerebrospinal fluid, serum biomarkers, longitudinal study, precision medicine

## Abstract

Tofersen is a gene-targeted therapy for superoxide dismutase 1 (*SOD1*)-associated amyotrophic lateral sclerosis (ALS), but neurofilament light chain (NfL) may not fully capture the biological response to treatment. We performed a multicentre retrospective longitudinal study including 24 patients with *SOD1*-ALS treated with intrathecal tofersen at four Italian referral centres between 2022 and 2025. Cerebrospinal fluid (CSF) and serum biomarkers were assessed at baseline, month 3, month 6, and last available administration using single-molecule array assays to quantify NfL, glial fibrillary acidic protein (GFAP), ubiquitin C-terminal hydrolase L1 (UCHL–1), and total Tau. NfL decreased after treatment initiation in both CSF and serum, providing the clearest pharmacodynamic signal. In contrast, CSF GFAP increased progressively over follow-up, while CSF total Tau and UCHL–1 rose mainly at later timepoints; serum GFAP, total Tau, and UCHL–1 also showed increases during follow-up. ALS Functional Rating Scale–Revised trajectories were broadly stable, whereas disease progression rate was lower at last follow-up than at baseline. Greater reductions in CSF NfL were observed in pathogenic versus uncertain *SOD1* variants, and early serum NfL and UCHL–1 changes were associated with longer-term changes in disease progression. These findings suggest that longitudinal multi-analyte profiling may refine biological response stratification beyond NfL alone in tofersen-treated *SOD1*-ALS.

## 1. Introduction

Tofersen has ushered in a new era of gene-targeted therapy for amyotrophic lateral sclerosis (ALS) caused by pathogenic variants in the superoxide dismutase 1 (*SOD1*) gene [1]. By binding *SOD1* mRNA and promoting RNase H-mediated degradation [2], this intrathecally administered antisense oligonucleotide reduces *SOD1* protein expression and directly targets toxic gain-of-function mechanisms implicated in *SOD1*-ALS [3,4].

In the VALOR trial, tofersen produced clear target engagement, reflected by reduced cerebrospinal fluid (CSF) *SOD1*, together with a reduction in plasma neurofilament light chain (NfL), providing the clearest biomarker signal of biological response to treatment in *SOD1*-ALS [1]. NfL has been consistently associated with disease aggressiveness, subsequent functional decline, and survival [5,6,7,8]. Its relative stability over short intervals in untreated disease, together with its sensitivity to therapeutic intervention, supports its use as a pharmacodynamic readout in ALS trials [9]. Longer-term follow-up of tofersen-treated *SOD1*-ALS patients reinforced NfL as a robust pharmacodynamic marker [10], and consistent neurofilament responses have also been reported in real-world cohorts [11,12,13]. Nevertheless, although NfL provides the most established readout of neuroaxonal injury and treatment-related pharmacodynamic response, it captures only one dimension of ALS biology. Additional biomarkers are therefore needed to reflect parallel processes that may shape disease course and therapeutic response [14,15].

ALS involves a complex interplay of neuroaxonal injury, glial reactivity, and neuroinflammation that may not be fully captured by NfL alone [16,17]. In addition, treatment-associated or procedure-associated immune activation (e.g., pleocytosis and/or intrathecal immunoglobulin synthesis) has emerged as a relevant consideration in intrathecal antisense oligonucleotide programmes, motivating close monitoring of glial and inflammatory signals alongside neurofilaments [13]. In parallel, neuronal injury markers beyond neurofilaments may add mechanistic resolution to treatment monitoring [18,19,20,21].

We therefore extended biomarker assessment beyond neurofilaments by including glial fibrillary acidic protein (GFAP), ubiquitin C-terminal hydrolase L1 (UCHL–1), and total Tau as complementary readouts. GFAP, the principal intermediate filament protein of mature astrocytes, is widely used as a fluid marker of astroglial pathology and reactive astrogliosis-associated processes [22,23]. Although GFAP has been extensively investigated in Alzheimer’s disease, multiple sclerosis, and other inflammatory or neurodegenerative disorders, it is not disease-specific, but rather reflects astrocyte responses to chronic tissue injury and neurodegeneration [24,25,26]. In ALS, reactive astrogliosis is a prominent feature of the motor cortex and spinal cord and contributes to non-cell-autonomous neurodegeneration [27,28,29,30,31]. Recent work has also suggested that the relationship between GFAP and NfL may provide information beyond either analyte alone, as their ratio discriminated ALS from Tau-related Progressive Supranuclear Palsy better than single analyte [32]. Although such composite ratios have not been established as treatment-response biomarkers in ALS, we explored if the NfL/GFAP ratio may provide additional information during targeted *SOD1* suppression. UCHL–1 is a neuron-enriched deubiquitinating enzyme implicated in ubiquitin-dependent proteostasis, regulation of the free ubiquitin pool, protein degradation, redox homeostasis, and axonal maintenance [33]. Increased CSF and serum UCHL–1 levels have been reported in ALS [20]. Total Tau, classically used in Alzheimer’s disease and Tauopathies, is a microtubule-associated neuronal protein with broader roles in cytoskeletal stability, axonal transport, synaptic function, and neuronal survival [34,35]. In ALS, CSF total Tau has been reported to have diagnostic and prognostic relevance, including associations with disease progression and survival [18].

Tofersen offers a unique opportunity to study the in vivo biological response to a causal, gene-targeted intervention in *SOD1*-ALS. By enabling longitudinal monitoring of multiple biofluid biomarkers, it allows characterisation of treatment-linked changes beyond clinical measures alone and supports a more granular understanding of downstream consequences of *SOD1* suppression. Defining tofersen-responsive biomarker patterns may strengthen biomarker endpoints and improve stratification of biological responders, refining interpretation of therapeutic engagement and informing next-generation *SOD1*-targeted strategies. Here, we present a longitudinal biomarker study in a multicentre Italian cohort of *SOD1*-ALS patients receiving tofersen, quantifying NfL, GFAP, UCHL–1, and total Tau in CSF and serum to delineate their temporal trajectories and evaluate whether multi-biomarker profiling provides added biological insight beyond neurofilaments alone.

## 2. Results

### 2.1. Study Cohort and Sample Availability

A total of 24 patients treated with tofersen were included in the biomarker analyses (Table 1). Females accounted for 13/24 (54%), mean age at onset was 50.8 years, and classic ALS was the most frequent phenotype (10/22, 45.4%). Across the cohort, *SOD1* variants spanned different pathogenicity classes: 6/24 (25%) were classified as variants of uncertain significance (VUS), 9/24 (37.5%) as likely pathogenic, and 9/24 (37.5%) as pathogenic (Table 2). The most frequent variant was c.435G>C (p.Leu145Phe), identified in four unrelated individuals; c.272A>C (p.Asp91Ala) was observed in three cases, two heterozygous and one homozygous. Notably, one of the heterozygous patients had a concomitant likely pathogenic variant in the *TARDBP* gene (p.Asn267Ser).

### 2.2. Baseline Associations Between Biomarkers and Clinical Variables

At baseline, biomarker levels showed several associations with clinical variables (Table S1). CSF NfL correlated positively with baseline DPR (ρ = 0.72, *p* < 0.001; n = 23) and inversely with respiratory support timing (NIV from onset: ρ = −0.78, *p* < 0.001; n = 18). Serum NfL showed a similar pattern, correlating with baseline DPR (ρ = 0.77, *p* < 0.001; n = 18) and inversely with NIV timing (ρ = −0.54; *p* = 0.038; n = 15). Serum GFAP correlated with age (ρ = 0.56; *p* = 0.016; n = 18). Serum UCHL–1 correlated inversely with baseline functional status (ALSFRS–R at T0: ρ = −0.55, *p* = 0.019; n = 18).

### 2.3. Clinical Trajectories After Tofersen Administration

**Figure 1:**
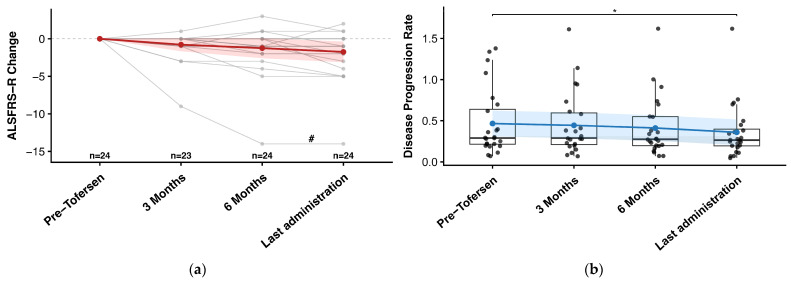
(**a**) Individual ALSFRS–R trajectories during tofersen treatment. Spaghetti plot of patient-level ALSFRS–R change relative to baseline across study visits. Grey lines represent individual patients; the red line indicates the cohort-level mean trend, with the shaded band showing the 95% confidence interval. (**b**) Disease progression rate during tofersen treatment. Box-and-whisker plots with overlaid individual data (black points) show disease progression rate (DPR) across study visits. Boxes indicate the interquartile range with the median line; whiskers extend to the most extreme values within 1.5 × IQR. Blue points and line indicate the cohort-level mean trend, with the shaded band showing the 95% confidence interval; * = *p* < 0.05; # = concomitant *TARDBP* variant (p.Asn267Ser).

Individual ALSFRS–R trajectories are shown in Figure 1 and suggest overall functional stability during follow-up. In contrast, DPR showed a timepoint effect in the mixed-effects model with a lower DPR at last administration (LA) versus T0 (*p* = 0.023; Figure 1). Over the observation period, two patients moved from the fast to the intermediate DPR category, one from the intermediate to the slow category, and one from the fast to the slow category (Figure S1). The only patient who continued to show a fast progression trajectory, without evidence of slowing over follow-up, carried a concomitant likely pathogenic *TARDBP* variant.

### 2.4. Longitudinal Biomarker Trajectories

**Figure 2:**
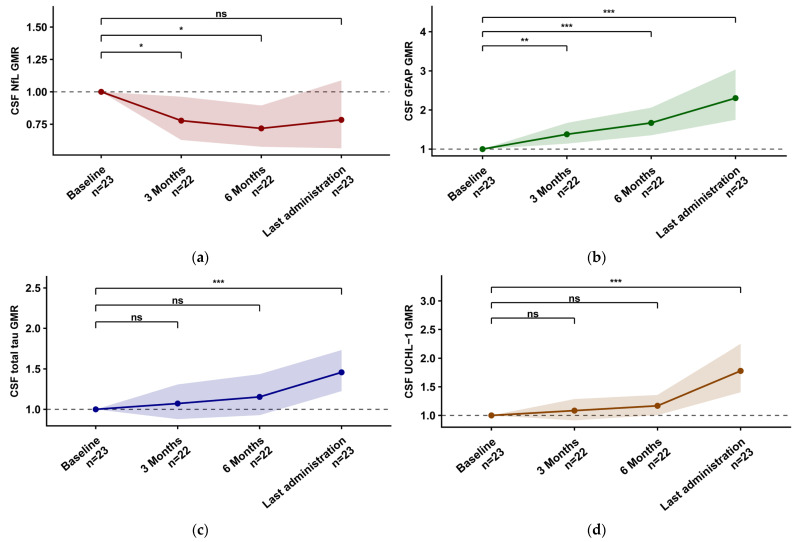
Longitudinal changes in CSF biomarkers during tofersen treatment. Geometric mean ratios (GMRs) versus baseline (Tx/T0) for CSF biomarkers are shown across study visits. Points and lines indicate GMRs, and shaded bands represent 95% confidence intervals. Significance is indicated as * = *p* < 0.05, ** = *p* < 0.01, *** = *p* < 0.001, ns = not significant. Abbreviations: CSF, cerebrospinal fluid; NfL, neurofilament light chain; GFAP, glial fibrillary acidic protein; UCHL–1, ubiquitin C-terminal hydrolase L1; GMR, geometric mean ratio. (**a**) CSF NfL, (**b**) CSF GFAP, (**c**) CSF total Tau, (**d**) CSF UCHL–1.

**Figure 3:**
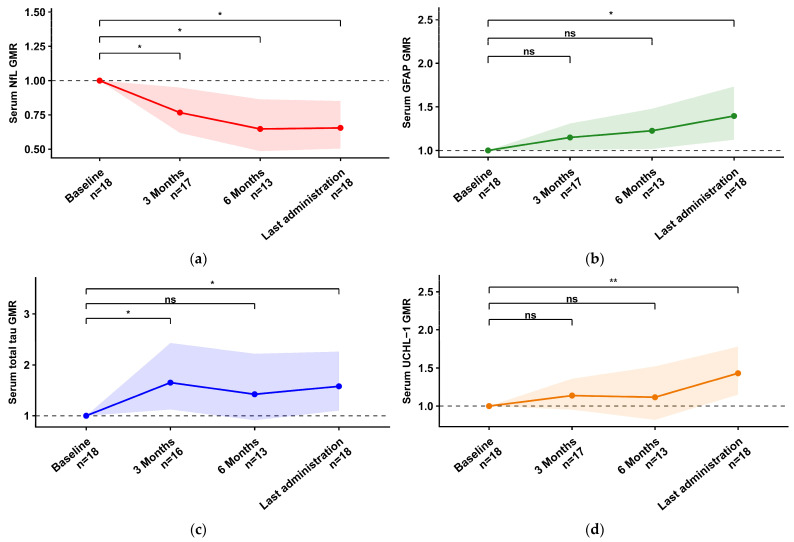
Longitudinal changes in serum biomarkers during tofersen treatment. Geometric mean ratios (GMRs) versus baseline (Tx/T0) for serum biomarkers are shown across study visits. Points and lines indicate GMRs, and shaded bands represent 95% confidence intervals. Significance is indicated as * = *p* < 0.05, ** = *p* < 0.01, ns = not significant. Abbreviations: NfL, neurofilament light chain; GFAP, glial fibrillary acidic protein; UCHL–1, ubiquitin C-terminal hydrolase L1; GMR, geometric mean ratio. (**a**) Serum NfL, (**b**) serum GFAP, (**c**) serum total Tau, (**d**) serum UCHL–1.

Baseline concentrations, longitudinal trajectories, and the number of evaluable samples are summarised in Table 3. Baseline correlations between biomarkers are reported in Table S2. After tofersen initiation, CSF NfL showed a consistent decline, reaching significance at T3 versus T0 (−22.3%; GMR 0.78, 95% CI 0.63–0.96; Holm-adjusted *p* = 0.046) and T6 versus T0 (−28.2%; GMR 0.72, 95% CI 0.58–0.89; Holm-adjusted *p* = 0.015). By contrast, CSF GFAP increased early and progressively, already higher at T3 (+37.9%; GMR 1.38, 95% CI 1.14–1.67; Holm-adjusted *p* = 0.002) and further rising at T6 (+67.0%; GMR 1.67, 95% CI 1.35–2.06; Holm-adjusted *p* < 0.001) and LA (+130.3%; GMR 2.30, 95% CI 1.75–3.03; Holm-adjusted *p* < 0.001). Consistent with these CSF trajectories, the CSF NfL/GFAP ratio decreased significantly over follow-up, already lower at T3 (−43.6%; GMR 0.56, 95% CI 0.46–0.69; Holm-adjusted *p* < 0.001), with further decreases at T6 (−57.0%; GMR 0.43, 95% CI 0.32–0.57; Holm-adjusted *p* < 0.001) and LA (−66.0%; GMR 0.34, 95% CI 0.23–0.50; Holm-adjusted *p* < 0.001) (Table S5, Figure S4). CSF total Tau and CSF UCHL–1 showed their main signal at LA, with significant increases versus baseline (CSF total Tau +45.8%; GMR 1.46, 95% CI 1.23–1.73; Holm-adjusted *p* < 0.001; CSF UCHL–1 +77.8%; GMR 1.78, 95% CI 1.41–2.25; Holm-adjusted *p* < 0.001) (Figure 2). In serum, NfL decreased at all follow-up timepoints (T3 −23.3%; GMR 0.77, 95% CI 0.62–0.95; Holm-adjusted *p* = 0.018; T6 −35.2%; GMR 0.65, 95% CI 0.49–0.86; Holm-adjusted *p* = 0.013; LA −34.5%; GMR 0.66, 95% CI 0.50–0.85; Holm-adjusted *p* = 0.010). Serum GFAP increased, reaching significance at LA (+39.5%; GMR 1.40, 95% CI 1.12–1.73; Holm-adjusted *p* = 0.015). Similarly, the serum NfL/GFAP ratio decreased at T3 (−33.3%; GMR 0.67, 95% CI 0.54–0.82; Holm-adjusted *p* < 0.001), T6 (−47.2%; GMR 0.53, 95% CI 0.43–0.65; Holm-adjusted *p* < 0.001), and LA (−53.0%; GMR 0.47, 95% CI 0.37–0.60; Holm-adjusted *p* < 0.001) (Table S5, Figure S4). Serum total Tau showed an early rise at T3 (+65.2%; GMR 1.65, 95% CI 1.12–2.43; Holm-adjusted *p* = 0.042) and remained higher at LA (+57.9%; GMR 1.58, 95% CI 1.10–2.26; Holm-adjusted *p* = 0.042), while serum UCHL–1 increased significantly at LA (+43.1%; GMR 1.43, 95% CI 1.15–1.78; Holm-adjusted *p* = 0.009) (Figure 3). CSF total protein data were available in eight patients. In timepoint-specific analyses, CSF total protein and CSF GFAP changes at T6/T0 showed a significant correlation (Spearman ρ = 0.964, n = 7, *p* = 0.009). No significant associations were observed for other CSF and serum biomarkers. In longitudinal mixed-effects models, CSF total protein changes were not significantly associated with CSF or serum biomarker ratios (Table S6, Figure S5).

### 2.5. Genotype–Biomarker Analysis

**Figure 4:**
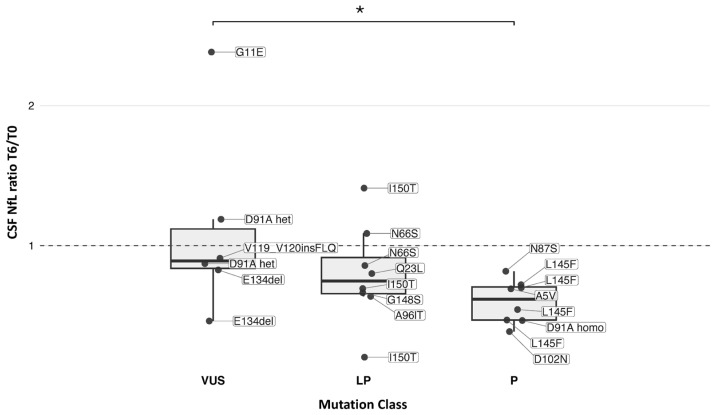
Six-month change in CSF NfL stratified by *SOD1* variant pathogenicity class. Box-and-whisker plots with overlaid individual data points show the CSF NfL ratios at T6/T0 stratified by variant class (VUS, LP, and P). Each point represents one patient and is annotated by the corresponding *SOD1* variant, central line denotes the median, boxes indicate the interquartile range (IQR), whiskers extend to values within 1.5 × IQR. Significance is indicated as * = *p* < 0.05. Abbreviations: VUS, variant of uncertain significance; LP, likely pathogenic; P, pathogenic; CSF, cerebrospinal fluid; NfL, neurofilament light chain.

We assessed whether the 6-month biomarker change (T6/T0) differed according to ACMG/AMP variant class (VUS, likely pathogenic, pathogenic). For CSF NfL ratio T6/T0, pairwise testing showed a difference between VUS and pathogenic variants (*p* = 0.033) (Figure 4). No class-related differences were observed for CSF GFAP, CSF total Tau, or CSF UCHL–1 at T6/T0, and no differences were evident for serum biomarkers.

### 2.6. Early Biomarker Changes as Predictors of Long-Term DPR Change

**Figure 5:**
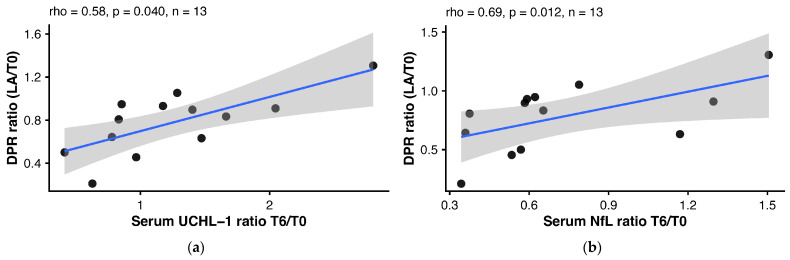
Early 6-month serum biomarker ratios and long-term DPR change. Panel plots show the association between early serum biomarker ratios at month 6 relative to baseline and DPR ratio change from baseline to LA. Panels show univariate associations for (**a**) Serum UCHL–1 ratio T6/T0 and (**b**) Serum NfL ratio T/T0, respectively. Points represent individual patients; solid lines indicate linear regression fits, with shaded bands showing 95% confidence intervals. Panel subtitles report Spearman’s rho, *p* value, and sample size (n). Abbreviations: DPR, disease progression rate; T0, baseline; LA, last administration/last available timepoint; NfL, neurofilament light chain; UCHL–1, ubiquitin C-terminal hydrolase L1.

To assess whether early biomarker changes predicted longer-term clinical evolution, we tested associations between T6/T0 ratios and last-observation outcomes. The strongest signal was for serum NfL T6/T0, which correlated with DPR ratio (LA/T0) (ρ = 0.69; 0.012; n = 13), indicating that a larger reduction in serum NfL ratio at T6 was associated with a greater reduction in DPR over follow-up. Consistent with this, serum NfL/GFAP T6/T0 correlated positively with DPR ratio (LA/T0) (ρ = 0.55; *p* = 0.049; n = 13), with a similar association observed for CSF NfL/GFAP T6/T0 (ρ = 0.48; *p* = 0.025; n = 22). However, these associations were weaker than that observed for NfL alone. An association was also observed for serum UCHL–1 T6/T0 (ρ = 0.58; *p* = 0.040; n = 13). We therefore fitted a multivariable linear regression model with DPR ratio between LA and T0 as the dependent variable and serum NfL T6/T0 and serum UCHL–1 T6/T0 ratios as mutually adjusted predictors (Figure 5). In univariable models (n = 13), both predictors were associated with DPR ratio (serum UCHL–1 T6/T0: β 0.318, 95% CI 0.113–0.524; *p* = 0.006; R^2^ = 0.513; serum NfL T6/T0: β 0.449, 95% CI 0.032–0.866; *p* = 0.037; R^2^ = 0.338). In the multivariable model, the associations were attenuated due to collinearity between predictors (Spearman ρ = 0.86, *p* < 0.001; VIF 4.14) (Table S4). The overall model remained significant and explained 52.1% of the variance in DPR ratio (R^2^ = 0.521; adjusted R^2^ = 0.425; overall model *p* = 0.025). In a complementary exploratory stratified analysis based on the median DPR LA/T0 ratio, serum NfL and serum UCHL–1 showed differential longitudinal trajectories between patients with greater versus lesser DPR reduction, as supported by significant group-by-time interactions (serum NfL, *p* = 0.0289; serum UCHL–1, *p* = 0.0379). Between-group differences emerged at T6 for both biomarkers (serum NfL, *p* = 0.0162; serum UCHL–1, *p* = 0.0138), and persisted at last administration for serum NfL (*p* = 0.0025) (Figure S2).

### 2.7. Early Biomarker Responder Subgroup Analysis

**Figure 6:**
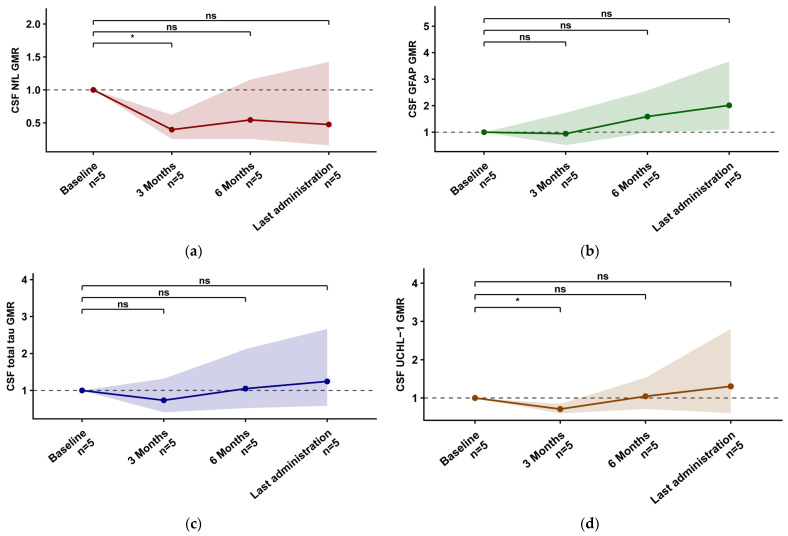
Longitudinal changes in CSF biomarkers during tofersen treatment in early responders. Geometric mean ratios (GMRs) versus baseline (Tx/T0) for serum biomarkers are shown across study visits. Points and lines indicate GMRs, and shaded bands represent 95% confidence intervals. Significance is indicated as * = *p* < 0.05, ns = not significant. Abbreviations: CSF, cerebrospinal fluid; NfL, neurofilament light chain; GFAP, glial fibrillary acidic protein; UCHL–1, ubiquitin C-terminal hydrolase L1; GMR, geometric mean ratio; (**a**): CSF NfL, (**b**): CSF GFAP, (**c**): CSF total Tau, (**d**): CSF UCHL–1.

In the early biomarker responder subgroup defined as the lowest tertile of the CSF NfL T3/T0 ratio, biomarker trajectories did not show different behaviours compared to those described in the global cohort (Table S3, Figure S3). In the early biomarker responder subgroup defined as the lowest quintile of the CSF NfL T3/T0 ratio, biomarker trajectories showed a distinct pattern compared with the overall cohort: CSF UCHL–1 showed an early reduction at T3 (GMR 0.71, 95% CI 0.59–0.85; Holm-adjusted *p* = 0.017), followed by a non-significant increase at later timepoints (Figure 6, Table 4). CSF total Tau displayed a qualitatively similar early downward trend, although not reaching statistical significance.

## 3. Discussion

In this multicentre real-world cohort of patients with *SOD1*-ALS treated with tofersen, ALSFRS–R trajectories were broadly stable over follow-up, whereas DPR decreased at LA, supporting a long-term signal consistent with slowing of clinical deterioration. At the biomarker level, we observed a coherent reduction in NfL in both CSF and serum after treatment initiation, alongside increasing GFAP in both compartments and later increases in CSF total Tau and CSF UCHL–1.

The decline in NfL in both CSF and serum supports a biologically coherent treatment effect consistent with prior tofersen evidence. VALOR and its extension demonstrated that tofersen produces clear target engagement and a marked decrease in plasma NfL, establishing NfL as the most robust pharmacodynamic biomarker in this setting [1,10]. Real-world cohorts have reproduced this neurofilament response, strengthening generalizability beyond the trial context [11,12,13]. Our results extend prior evidence by confirming a consistent reduction in NfL during tofersen treatment.

While this supports NfL as a reliable pharmacodynamic marker of treatment engagement, the interpretation of NfL changes is nuanced by the observation that other neuronal injury biomarkers—such as UCHL–1 and total Tau—do not follow an equally linear trajectory. This divergence suggests that equating neurofilament release into biofluids with the degree and rate of axonal destruction alone may be an oversimplification [36]. Indeed, it has been proposed that increased neurofilament expression in ALS may also reflect an adaptive, energy-saving shift in motor neurons, with preferential expression of smaller, less energy-demanding neurofilament subunits under conditions of heightened metabolic stress [37]. In this framework, longitudinal changes in NfL may capture not only structural axonal breakdown or rescue, but also a broader and multifaceted neuronal response to neurodegeneration, limiting its interpretation as a proxy of clinical response in the real-world setting.

In contrast to decreasing NfL, GFAP increased progressively in CSF and serum, suggesting that astroglial pathology may follow a partially independent trajectory during tofersen treatment. However, fluid GFAP should not be interpreted as a direct readout of astrocyte activation alone, since increased concentrations may reflect reactive astrogliosis, astrocytic injury, protein release, blood–brain barrier permeability, peripheral distribution, and systemic clearance [22,23].

This interpretation is aligned with an emerging tofersen literature reporting inflammatory CSF findings—including pleocytosis and intrathecal immunoglobulin synthesis—during follow-up in real-world cohorts [13]. Consistently, in our cohort, CSF GFAP was the biomarker most closely related to routine CSF total protein changes.

Clinically, higher baseline NfL was associated with higher baseline DPR and earlier NIV timing, consistent with NfL reflecting disease aggressiveness at treatment initiation. Serum UCHL–1 correlated inversely with baseline ALSFRS–R, supporting the idea that neuronal proteins beyond neurofilaments may capture complementary information about disease state.

Unlike the trial setting, where enrolment was enriched for patients carrying *SOD1* variants associated with a more aggressive disease course [1], real-world cohorts inevitably include a broader and more heterogeneous genetic spectrum, including VUS. This is particularly relevant for variants such as p.Asp91Ala in the heterozygous state, whose pathogenic role may be uncertain in individual cases [38]. In this real-world context, neurofilament dynamics might provide clinically useful supportive evidence for variant interpretation, helping to clarify whether a given variant is truly disease-causing when the genetic finding alone is insufficient. In our genotype–biomarker analyses, CSF NfL was the main analyte showing a differential short-term response across variant categories, with pathogenic variants showing a larger 6-month reduction than VUS. Although this comparison should be interpreted cautiously, it is biologically plausible: a stronger neurofilament response would be expected when the underlying aetiology is truly *SOD1*-driven, whereas the VUS category is inherently heterogeneous and may include variants with uncertain pathogenic contribution and where oligogenicity should be considered. This point is exemplified by the patient carrying heterozygous p.Asp91Ala, who also harboured a likely pathogenic *TARDBP* variant, highlighting how, in real-world practice, a more refined selection of candidates for gene-targeted therapies may be needed to ensure that treatment is directed toward cases in which *SOD1* is the most plausible disease driver.

We next examined heterogeneity of early biological response using a responder definition anchored to early CSF NfL change. At the cohort level, UCHL–1 showed a more complex course than NfL, with modest early CSF changes and larger late increases, and limited interpretability in serum given lower availability. The early biomarker responder analysis provides an important refinement: among responders in the lowest quintile of NfL reduction, CSF UCHL–1 decreased significantly at 3 months. This responder-enriched CSF signal is concordant with proteomic evidence indicating that UCHL–1 can behave as an early tofersen-responsive neuronal marker, with decreases at early timepoints [39]. Notably, Steffke and colleagues also observed that this early UCHL–1 reduction may not persist with longer follow-up as inflammatory pathways become more prominent, suggesting a time-dependent or biphasic behaviour. From a mechanistic perspective, this pattern may suggest that, in biologically responsive patients, tofersen is associated not only with reduced structural axonal injury, as captured by NfL, but also with an early attenuation of neuronal proteostatic stress and injury-related ubiquitin–proteasome pathway activation, as reflected by UCHL–1. Our results partially fit this model and support the hypothesis that UCHL–1 reduction may identify a subset with stronger early biological response, whereas longer-term trajectories may be modulated or obscured by competing inflammatory or degenerative processes.

Taken together with the GFAP rise observed in our cohort, these data reinforce the concept that longer-term treatment exposure may be associated with an evolving CSF inflammatory milieu that is not mirrored by neurofilament behaviour and might warrant further investigation, potentially modulating long-term biomarker trajectories [40].

The divergent behaviour of total Tau and NfL should be interpreted cautiously. CSF total Tau is an assay-defined biomarker that may capture biological processes partly distinct from the axonal structural injury indexed by NfL. Although Tau has traditionally been interpreted as a marker of neuronal injury, recent evidence suggests that CSF total Tau is closely associated with synaptic biomarkers, supporting its potential role as a proxy of synaptic dysfunction or degeneration rather than a pure marker of axonal loss [41]. Experimental data also indicate that neuronal activity can regulate extracellular Tau levels, and that Tau participates in synaptic physiology and dysfunction, providing a plausible biological framework for Tau changes that are not fully mirrored by neurofilament dynamics [42,43,44]. Thus, the increase in total Tau during continued intrathecal therapy may reflect persistent synaptic stress, activity-dependent neuronal Tau secretion, or altered extracellular Tau processing, rather than ongoing axonal degeneration alone. In this context, the parallel increase in GFAP may indicate that Tau dynamics occur within a CSF milieu increasingly influenced by astroglial and inflammatory changes; however, a direct astroglial origin of Tau should be regarded only as a possible contributing mechanism, rather than the primary interpretation [45,46].

Finally, analyses linking early biomarker changes to longer-term clinical evolution identified serum NfL and UCHL–1 reduction at T6 as the most informative early correlates of DPR change at last administration. These findings support the clinical relevance of early serum NfL and UCHL–1 dynamics and suggest that the depth of biomarker reduction may stratify longer-term progression trajectories during tofersen therapy, warranting validation in larger datasets.

## 4. Materials and Methods

### 4.1. Study Design and Cohort

We performed a multicentre, retrospective cohort study including 24 individuals with *SOD1*-associated ALS who received tofersen between 2022 and 2025 at four Italian ALS referral centres (IRCCS Fondazione Istituto Neurologico Carlo Besta; Istituti Clinici Maugeri, Milan; NeMo Clinical Center, Milan; and University of Modena and Reggio Emilia). Clinical data and biospecimens (CSF and, when available, serum) were collected longitudinally at approximately 3-month intervals. None of the included patients participated in the VALOR trial.

Tofersen was administered intrathecally at 100 mg according to the Early Access Program schedule (loading doses on days 1, 14, and 28, followed by maintenance dosing every 28 days). Baseline (T0) was defined as the visit corresponding to the first intrathecal administration. Follow-up timepoints were defined as T3 (mean 89.5 [SD 12.0] days from baseline; range 69–118), T6 (mean 177.0 [SD 13.3] days; range 131–198), and last administration (LA; mean 379.5 [SD 168.3] days; range 168–753), corresponding to the last available visit within the observation window. The study was conducted in accordance with the Declaration of Helsinki and received approval from the local Ethics Committee.

### 4.2. Clinical Variables and Outcomes

At baseline, we recorded sex, age at symptom onset, diagnostic delay, family history of ALS and/or frontotemporal dementia, site of onset (bulbar, upper limb, lower limb, respiratory), and clinical phenotype (classic, bulbar, upper motor neuron-predominant, flail arm, flail leg, respiratory, or progressive muscular atrophy presentation) [47,48]. Anthropometrics (height, weight) were collected; baseline weight loss was defined as the difference (kg) between premorbid weight (before symptom onset) and weight at T0. Respiratory function was evaluated by forced vital capacity (FVC) [49].

At each tofersen administration visit, functional status was assessed using the ALS Functional Rating Scale–Revised (ALSFRS–R). Disease progression rate (DPR) was calculated at each timepoint as (48 − ALSFRS–R) divided by disease duration in months from symptom onset to the assessment. A classification of DPR of slower ALS (<0.5 ALSFRS–R/month), intermediate ALS (≥0.5 and ≤1.0 ALSFRS–R/month) and faster ALS (>1.0 ALSFRS–R/month) progression was applied [50]. DPR pre-treatment was calculated from the time of onset to the initiation of tofersen treatment. DPR during treatment described ALS progression rate from the time of onset to each timepoint of tofersen administration.

### 4.3. Genetic Annotation

*SOD1* variants were annotated for each patient by coding change and predicted protein change, together with genomic location (exon, or intronic/5′UTR when applicable). Variants were classified according to the American College of Medical Genetics and Genomics and the Association for Molecular Pathology (ACMG/AMP) standards and guidelines as pathogenic, likely pathogenic, or a variant of uncertain significance (VUS) [51]. When available, in silico pathogenicity metrics were reported, including the Combined Annotation-Dependent Depletion (CADD) score [52] and the Rare Exome Variant Ensemble Learner (REVEL) score [53].

### 4.4. Biospecimen Collection and Biomarker Assays

Serum and cerebrospinal fluid were collected by venipuncture and lumbar puncture as part of the tofersen administration protocol, processed according to standardised pre-analytical procedures, aliquoted into polypropylene tubes, and stored at −80 °C until analysis. Samples were centralised and analysed at the IRCCS Fondazione Istituto Neurologico Carlo Besta laboratory. Concentrations of NfL, GFAP, UCHL–1, and total Tau were measured in duplicate using a multiplex (4-plex) single-molecule array digital immunoassay (Simoa; Quanterix, Billerica, MA, USA). The mean of the two replicate concentrations was used for all analyses. Assays were run with manufacturer-recommended calibrators and internal quality controls, and laboratory personnel were blinded to clinical data [54]. Biomarkers were evaluated at predefined timepoints (T0, T3, T6, and LA). No additional post hoc filtering based on coefficient of variation was applied, and missing values were not imputed [55]. Routine CSF total protein values, when available, were retrieved from clinical records at the corresponding tofersen administration visits. CSF cell-count data were not systematically available across centres and timepoints.

### 4.5. Statistical Analysis

Descriptive statistics are presented as mean (SD) for approximately normally distributed variables or median (IQR) for skewed distributions, and as number (%) for categorical variables. All analyses were performed on available data, no imputation for missing data was applied.

Longitudinal biomarker changes were summarised as within-participant ratios to baseline (Tx/T0). For group-level inference, ratio changes were analysed on the log scale: for each biomarker and timepoint (T3, T6, and LA), we calculated geometric mean ratios (GMRs) using paired observations at both timepoints and derived 95% confidence intervals (CIs). Departure of the GMR from 1 was tested using one-sample *t* tests on log(Tx/T0); *p* values across follow-up timepoints were adjusted using the Holm method. NfL/GFAP ratios were additionally calculated in serum and CSF, and their baseline clinical associations, longitudinal Tx/T0 changes, and correlations with longer-term clinical outcomes were analysed using the same analytical framework applied to the individual biomarkers. Available CSF total protein values were analysed using the same ratio-based framework applied to CSF biomarkers. Within-participant ratios to baseline were calculated for each post-baseline timepoint when paired data were available. Timepoint-specific associations between CSF total protein and CSF biomarker ratios were assessed using Spearman’s rank correlation coefficient, with Holm correction across post-baseline timepoints. Longitudinal associations between CSF total protein changes and biomarker changes were further assessed using linear mixed-effects models, with log-transformed biomarker ratio as the dependent variable, log-transformed CSF total protein ratio and timepoint as fixed effects, and participant-specific random intercepts.

We used two sequential approaches to identify an early responder subgroup based on early CSF NfL decline: first, participants were stratified into tertiles according to the CSF NfL T3/T0 ratio; then, a stricter definition was applied using the lowest quintile of the same ratio, corresponding to those with the greatest early CSF NfL reduction, to assess whether the other biomarkers showed a distinct longitudinal profile consistent with prior proteomic responder-stratification approaches (Steffke et al., 2025) [39].

Between-group comparisons were performed using non-parametric tests (Mann–Whitney U for two groups; Kruskal–Wallis for three or more groups), with post hoc pairwise Wilcoxon tests used where appropriate. Associations between continuous variables (baseline measures and ratio changes) were assessed using Spearman’s rank correlation coefficient. Differences in biomarker ratio changes across *SOD1* variant classes—classified according to the ACMG/AMP framework—were evaluated using Kruskal–Wallis and pairwise Wilcoxon tests.

DPR across timepoints (T0, T3, T6, and LA) was modelled using linear mixed-effects models with participant-specific random intercepts and time as a fixed effect; estimated marginal means and Holm-adjusted pairwise contrasts were obtained. Predictors of longer-term DPR change were primarily examined using uni- and multivariable linear regression models incorporating early biomarker ratio changes, with collinearity assessed using variance inflation factors. As a complementary exploratory approach, participants were additionally stratified according to the median DPR LA/T0 ratio (greater vs. lesser DPR reduction), and differences in longitudinal biomarker trajectories were assessed using linear mixed-effects models with group, timepoint, and their interaction as fixed effects and participant-specific random intercepts. Estimated marginal means were back-transformed and reported as geometric mean ratios (GMRs) with 95% confidence intervals; post hoc contrasts were Holm adjusted.

All analyses were conducted in R version 4.5.2 (R Foundation for Statistical Computing, Vienna, Austria). Statistical significance was defined as a two-sided *p* value < 0.05.

## 5. Limitations

This study has several limitations. First, the cohort size was modest and sample availability was incomplete and uneven across biomarkers, matrices, and timepoints—particularly in serum and after T6—reducing statistical power and yielding variable pairwise sample sizes across analyses. Second, the observational retrospective design and the use of an analysis-by-analysis complete-case approach could have introduced selection bias if missingness was not completely random.

In addition, subgroup and predictive analyses relied on small numbers. The responder subgroup, defined by the lowest quintile of CSF NfL change, included five individuals and should be considered exploratory. Similarly, the regression models linking early ratios to long-term DPR change were fitted on a limited complete-case subset, and estimates may be sensitive to influential observations. Finally, residual pre-analytical variability inherent to multicentre real-world collections cannot be fully excluded despite standardised handling and centralised testing.

## 6. Conclusions

In this multicentre real-world *SOD1*-ALS cohort treated with tofersen, NfL decreased consistently in CSF and serum, supporting reduced neuroaxonal injury as the clearest pharmacodynamic signature of *SOD1* suppression. In parallel, GFAP increased in both biofluids and CSF total Tau rose over follow-up, indicating that astroglial/inflammatory and broader neuronal injury pathways may evolve independently of the neurofilament response. The responder-enriched reduction in CSF UCHL–1 at 3 months suggests that neuronal proteins beyond NfL can add mechanistic resolution, although their trajectories differ between CSF and serum and may vary over time. Overall, these findings support longitudinal multi-analyte biomarker profiling to refine biological response stratification in tofersen-treated *SOD1*-ALS and to inform the optimisation of next-generation *SOD1*-targeted therapies.

## Figures and Tables

**Table 1:** Baseline demographic and clinical characteristics of the study cohort. Data are reported as N (%) for categorical variables and as mean ± SD for continuous variables, unless otherwise indicated. Abbreviations: ALSFRS–R: Amyotrophic Lateral Sclerosis Functional Rating Scale–Revised; DPR: disease progression rate, FVC: forced vital capacity (% predicted); SD, standard deviation; UL, upper limb; LL, lower limb; PLMN, pure lower motor neuron; PUMN, pure upper motor neuron; IQR, interquartile range.

Clinical Feature	Summary
Enrolled patients	N = 24
Sex	
Male	11/24 (46%)
Female	13/24 (54%)
Site of onset	N = 15
Proximal UL	5/15 (33.3%)
Distal LL	8/15 (53.3%)
Distal UL	2/15 (13.3%)
Weight at baseline (kg)	72.71 ± 12.42
Phenotype	
PLMN	3/22 (13.6%)
Classic	10/22 (45.5%)
Flail arm	2/22 (9.1%)
Flail leg	5/22 (22.7%)
Pyramidal	1/22 (4.5%)
PUMN	1/22 (4.5%)
Age at onset, years	50.82 ± 9.38
Age at diagnosis, years	52.61 ± 9.58
Age at baseline, years	56.77 ± 10.50
Months from onset to baseline, median [IQR]	58.79 [28.13–103.48]
ALSFRS–R at baseline, median [IQR]	29.50 [20.00–34.00]
DPR at baseline, median [IQR]	0.29 [0.21–0.66]
FVC (% predicted) at baseline, median [IQR]	65 [42–80]

**Table 2:** *SOD1* genotypes and variant-level annotations in the study cohort. The table reports, for each patient, the identified *SOD1* variant (coding change and predicted protein change), genomic location (exon/intronic/5′UTR when applicable), in silico prediction metrics (CADD and REVEL scores), and the assigned pathogenicity classification (pathogenic, likely pathogenic, or variant of uncertain significance). Patient-level clinical descriptors shown include age at onset, sex, phenotype, baseline ALSFRS–R during tofersen treatment, and DPR pre-tofersen and at last tofersen administration. Abbreviations: CADD, Combined Annotation-Dependent Depletion; REVEL, Rare Exome Variant Ensemble Learner; ALSFRS–R, Amyotrophic Lateral Sclerosis Functional Rating Scale–Revised; T0, baseline; LA, last administration; UTR, untranslated region; NA, not available. # = concomitant *TARDBP* variant (p.Asn267Ser).

Mutation	Exon	CADD	REVEL	ACGM	Age at Onset	Sex	Phenotype	ALSFRS–R at T0	DPR Pre-Tofersen	DPR at LA
p.Gly11Glu	5′UTR			VUS	61.7	F	Fail arm	26	Slow	Slow
c.14C>T (p. Ala5Val)	I	25.7	0.85	Pathogenic	27	F	Classic	38	Fast	Intermediate
c.68A>T (p.Gln23Leu)	I	23.5	0.79	Likely pathogenic	32.4	F	PUMN	22	Slow	Slow
c.197A>G (p.Asn66Ser)	III	26.1	0.94	Likely pathogenic	47.1	F	Classic	15	Slow	Slow
c.197A>G (p.Asn66Ser)	III	26.1	0.94	Likely pathogenic	56	F	Fail leg	16	Slow	Slow
p.Ser108Leufs*15	IV	NA	NA	Pathogenic	64.8	M	NA	16	Slow	Slow
c.260A>G (p. Asn87Ser)	IV	22.7	0.85	Pathogenic	58.8	F	PLMN	45	Slow	Slow
c.272A>C (p.Asp91Ala) Heterozygous #	IV	9.48	0.55	VUS	54.3	F	Pyramidal	34	Fast	Fast
c.272A>C (p.Asp91Ala) Heterozygous	IV	9.48	0.55	VUS	58.3	M	Classic	30	Slow	Slow
c.272A>C (p.Asp91Ala) Homozygous	IV	9.48	0.55	Pathogenic	59.2	M	Classic	34	Slow	Slow
c.286G>A (p.Ala96Thr)	IV	22.9	0.78	Likely pathogenic	45.3	M	PLMN	34	Slow	Slow
c.286G>A (p.Ala96Thr)	IV	22.9	0.776	Likely pathogenic	60.3	F	Classic	27	Slow	Slow
c.304G>A (p.Asp102Asn)	IV	25.3	0.83	Pathogenic	50.4	F	Classic	29	Fast	Slow
c.358-10 T>G	Intronic			VUS	49	M	Fail leg	33	Intermediate	Slow
p.Glu134del	V	NA	NA	VUS	53.4	F	Classic	34	Fast	Intermediate
p.Glu134del	V	NA	NA	VUS	61.6	F	Fail leg	33	Slow	Slow
c.435G>C (p.Leu145Phe)	V	24	0.91	Pathogenic	41.2	M	Classic	22	Slow	Slow
c.435G>C (p.Leu145Phe)	V	24	0.91	Pathogenic	43.3	M	Fail leg	40	Slow	Slow
c.435G>C (p.Leu145Phe)	V	24	0.91	Pathogenic	45.7	F	Classic	20	Slow	Slow
c.435G>C (p.Leu145Phe)	V	24	0.91	Pathogenic	49.9	M	Classic	18	Intermediate	Intermediate
c.442G>A (p.Gly148Ser)	V	26.3	0.969	Likely pathogenic	54.8	M	NA	14	Intermediate	Intermediate
c.449T>C (p.Ile150Thr)	V	26.6	0.968	Likely pathogenic	40.5	M	Fail arm	44	Slow	Slow
c.449T>C (p.Ile150Thr)	V	26.6	0.55	Likely pathogenic	50	M	Fail leg	20	Slow	Slow
C.449T>C (p.Ile150Thr)	V	26.6	0.96	Likely pathogenic	54.6	F	PLMN	39	Slow	Slow

**Table 3:** Baseline and longitudinal biomarker concentrations in serum and CSF during tofersen treatment. The table reports concentrations (pg/mL) of GFAP, NfL, UCHL–1, and total Tau measured in serum and cerebrospinal fluid (CSF) at baseline, month 3, month 6, and at the last administration/last available timepoint. For each analyte and timepoint, the number of evaluable samples (N) and summary statistics (median and IQR) are provided. Variations in N across biomarkers and timepoints reflect longitudinal sample availability. Abbreviations: CSF, cerebrospinal fluid; GFAP, glial fibrillary acidic protein; NfL, neurofilament light chain; UCHL–1, ubiquitin C-terminal hydrolase L1.

	Baseline	Month 3	Month 6	Last Administration
Serum				
UCHL–1 (pg/mL)	39.97 [24.97] (n = 18)	41.50 [27.64] (n = 17) GMRΔ +13.8%	44.66 [37.94] (n = 13) GMRΔ +11.6%	49.09 [40.30] (n = 18) GMRΔ +43.1%
NfL (pg/mL)	31.20 [29.23] (n = 18)	21.60 [14.28] (n = 17) GMRΔ −23.3%	18.45 [10.62] (n = 13) GMRΔ −35.2%	19.40 [14.80] (n = 18) GMRΔ −34.5%
GFAP (pg/mL)	137.72 [165.19] (n = 18)	147.95 [113.95] (n = 17) GMRΔ +14.9%	127.47 [154.88] (n = 13) GMRΔ +22.6%	174.80 [270.82] (n = 18) GMRΔ +39.5%
Total Tau (pg/mL)	0.42 [0.84] (n = 18)	0.97 [1.19] (n = 16) GMRΔ +65.2%	0.61 [1.07] (n = 13) GMRΔ +42.3%	0.76 [0.97] (n = 18) GMRΔ +57.9%
CSF				
UCHL–1 (pg/mL)	1828.72 [1231.43] (n = 23)	2158.38 [1235.20] (n = 22) GMRΔ +8.4%	2300.55 [1793.13] (n = 22) GMRΔ +16.8%	3105.21 [2194.22] (n = 23) GMRΔ +77.8%
NfL (pg/mL)	2156.56 [2816.57] (n = 23)	1986.47 [2112.00] (n = 22) GMRΔ −22.3%	1460.28 [2572.63] (n = 22) GMRΔ −28.2%	1652.33 [1621.83] (n = 23) GMRΔ −21.6%
GFAP (pg/mL)	5630.31 [7821.74] (n = 23)	7418.40 [12,733.05] (n = 22) GMRΔ +37.9%	9932.60 [11,416.80] (n = 22) GMRΔ +67.0%	15,250.40 [11,879.26] (n = 23) GMRΔ +130.3%
Total Tau (pg/mL)	58.83 [27.16] (n = 23)	69.41 [29.03] (n = 22) GMRΔ +7.2%	73.61 [26.93] (n = 22) GMRΔ +15.4%	80.47 [28.36] (n = 23) GMRΔ +45.8%

**Table 4:** Baseline and longitudinal biomarker concentrations in serum and CSF during tofersen treatment in the early biomarker responder subgroup (lowest quintile of NfL T3/T0 ratio). The table reports concentrations (pg/mL) of GFAP, NfL, UCHL–1, and total Tau measured in serum and cerebrospinal fluid (CSF) at baseline, month 3, month 6, and at the last administration/last available timepoint. For each analyte and timepoint, the number of evaluable samples (N) and summary statistics (median and IQR) are provided. Variations in N across biomarkers and timepoints reflect longitudinal sample availability. Abbreviations: GFAP, glial fibrillary acidic protein; NfL, neurofilament light chain; UCHL–1, ubiquitin C-terminal hydrolase L1.

	Baseline	Month 3	Month 6	Last Administration
Serum				
UCHL–1 (pg/mL)	42.83 [20.79] (n = 4)	39.83 [26.51] (n = 4) GMRΔ +8.1%	48.84 [56.61] (n = 3) GMRΔ +58.6%	57.41 [43.28] (n = 4) GMRΔ +67.1%
NfL (pg/mL)	57.24 [75.00] (n = 4)	27.20 [55.13] (n = 4) GMRΔ −19.6%	15.85 [91.62] (n = 3) GMRΔ −15.1%	26.12 [62.07] (n = 4) GMRΔ −34.0%
GFAP (pg/mL)	201.88 [186.64] (n = 4)	303.92 [276.54] (n = 4) GMRΔ +51.5%	413.76 [186.75] (n = 3) GMRΔ +63.9%	445.26 [143.26] (n = 4) GMRΔ +103.6%
Total Tau (pg/mL)	0.60 [0.41] (n = 4)	1.25 [1.94] (n = 4) GMRΔ +11.9%	0.61 [0.78] (n = 3) GMRΔ −21.1%	0.56 [0.39] (n = 4) GMRΔ −18.4%
CSF				
UCHL–1 (pg/mL)	3002.72 [1307.35] (n = 5)	2138.66 [1779.03] (n = 5) GMRΔ −29.0%	3105.21 [1533.11] (n = 5) GMRΔ +4.4%	4306.88 [3609.25] (n = 5) GMRΔ +30.5%
NfL (pg/mL)	4623.43 [5502.97] (n = 5)	1775.23 [1765.43] (n = 5) GMRΔ −60.3%	3211.90 [2545.60] (n = 5) GMRΔ −45.6%	3170.65 [2545.60] (n = 5) GMRΔ −52.4%
GFAP (pg/mL)	9342.73 [17,097.69] (n = 5)	11,274.85 [12,774.94] (n = 5) GMRΔ −5.7%	15,251.59 [21,494.28] (n = 5) GMRΔ +58.9%	22,772.54 [21,494.28] (n = 5) GMRΔ +101.2%
Total Tau (pg/mL)	72.62 [24.56] (n = 5)	56.20 [31.55] (n = 5) GMRΔ −26.8%	74.31 [26.77] (n = 5) GMRΔ +4.9%	95.53 [55.80] (n = 5) GMRΔ +24.4%

## Data Availability

The data that support the findings of this study are available from the corresponding author (N.R.), upon reasonable requests. The data are not publicly available due to privacy and ethical restrictions related to the inclusion of potentially identifiable clinical and biomarker information from patients with rare genetic variants.

## Supplementary Materials

The following supporting information can be downloaded at: https://www.mdpi.com/article/10.3390/ijms27104208/s1.

## Abbreviations

The following abbreviations are used in this manuscript:

ACMG/AMP	American College of Medical Genetics and Genomics/Association for Molecular Pathology
ALS	Amyotrophic Lateral Sclerosis
ALSFRS–R	Amyotrophic Lateral Sclerosis Functional Rating Scale–Revised
CADD	Combined Annotation-Dependent Depletion
CSF	Cerebrospinal Fluid
DPR	Disease Progression Rate
FVC	Forced Vital Capacity
GFAP	Glial Fibrillary Acidic Protein
NfL	Neurofilament Light Chain
NIV	Non-Invasive Ventilation
REVEL	Rare Exome Variant Ensemble Learner
RNase H	Ribonuclease H
*SOD1*	*Superoxide Dismutase 1*
*TARDBP*	*TAR DNA-Binding Protein*
UCHL–1	Ubiquitin C-Terminal Hydrolase L1
UTR	Untranslated Region
