# Supplementary material for: Longitudinal CSF and Serum Biomarker Dynamics in Tofersen-Treated SOD1-ALS: A Real-World Multicentre Cohort Study
Source: Int J Mol Sci. 2026 May 9;27(10):4208. doi: 10.3390/ijms27104208 (PMC13207959; doi:10.3390/ijms27104208)

Supplementary Materials

Longitudinal CSF and Serum Biomarker Dynamics in Tofersen-Treated *SOD1*-ALS: A Real-World Multicentre Cohort Study

Andrea Giordano [1,2], Jessica Mandrioli [3,4], Federica Cerri [5], Christian Lunetta [6], Hamidreza Saebfar [1], Marcella Catania [7], Claudia Battipaglia [7], Laura Leone [1], Francesca Trojsi [8], Maria Vizziello [1], Francesca Gerardi [5], Matteo Farè [5], Aida Zulueta [6], Rachele Piras [6], Matteo Giacchino [3,4], Giulia Gianferrari [3,4], Eleonora Dalla Bella [1], Teuta Domi [9], Dario Bonanomi [9], Giuseppe Ganci [10], Raffaella Lombardi [1], Giuseppe Lauria [1] and Nilo Riva [1,*]

[1] Neurology 3—Neuroalgology Unit, Fondazione IRCCS Istituto Neurologico Carlo Besta, Via Celoria 11, 20133 Milan, Italy
[2] Department of Medical Biotechnology and Translational Medicine, Università degli Studi di Milano, Via Vanvitelli 32, 20133 Milan, Italy
[3] Department of Neurosciences, Ospedale Civile Baggiovara, Azienda Ospedaliero Universitaria di Modena, Via Giardini 1355, 41126 Modena, Italy
[4] Department of Biomedical, Metabolic and Neural Sciences, University of Modena and Reggio Emilia, Via Campi 287, 41125 Modena, Italy
[5] NEuroMuscular Omnicentre, Fondazione Serena ETS, Piazza dell'Ospedale Maggiore, 3, 20162 Milan, Italy
[6] Neurorehabilitation Department, Istituti Clinici Scientifici Maugeri IRCCS, Via Camaldoli 64, 20138 Milan, Italy
[7] Neurology 8—Dementias and Degenerative Diseases of Central Nervous System Unit, Fondazione IRCCS Istituto Neurologico Carlo Besta, Via Celoria 11, 20133 Milan, Italy
[8] Neurology Unit, First Division of Neurology and Neurophysiopathology, AOU University of Campania "Luigi Vanvitelli", Piazza Miraglia 2, 80138 Naples, Italy
[9] Division of Neuroscience, IRCCS San Raffaele Scientific Institute, Via Olgettina 60, 20132 Milan, Italy
[10] Neurointerventional Radiology Unit, Fondazione IRCCS Istituto Neurologico Carlo Besta, Via Celoria 11, 20133 Milan, Italy
* Correspondence: nilo.riva@istituto-besta.it

Table S1. Baseline associations between biomarkers and clinical variables.

Biomarker	Age at onset	Age at diagnosis	Diagnosis-to-baseline interval	Weight at baseline	CADD score	REVEL score	SIFT score	PolyPhen-2 score	Time from onset to PEG	Time from onset to NIV	ALSFRS-R at baseline	DPR at baseline	FVC at baseline
**Serum GFAP at T0**	rho=0.684; p=0.002; n=18	rho=0.699; p=0.001; n=18	rho=0.071; p=0.779; n=18	rho=0.178; p=0.601; n=11	rho=-0.392; p=0.165; n=14	rho=0.130; p=0.657; n=14	rho=0.055; p=0.853; n=14	rho=-0.011; p=0.970; n=14	rho=0.321; p=0.482; n=7	rho=-0.061; p=0.830; n=15	rho=-0.090; p=0.722; n=18	rho=-0.010; p=0.967; n=18	rho=0.238; p=0.374; n=16
**Serum NfL at T0**	rho=-0.011; p=0.964; n=18	rho=-0.084; p=0.742; n=18	rho=-0.513; p=0.030; n=18	rho=0.009; p=0.979; n=11	rho=0.129; p=0.659; n=14	rho=-0.212; p=0.467; n=14	rho=-0.189; p=0.517; n=14	rho=-0.186; p=0.525; n=14	rho=-0.607; p=0.148; n=7	rho=-0.539; p=0.038; n=15	rho=-0.091; p=0.719; n=18	rho=0.774; p<0.001; n=18	rho=0.209; p=0.437; n=16
**Serum UCHL-1 at T0**	rho=0.416; p=0.086; n=18	rho=0.395; p=0.104; n=18	rho=0.261; p=0.295; n=18	rho=0.109; p=0.749; n=11	rho=-0.062; p=0.832; n=14	rho=-0.318; p=0.267; n=14	rho=0.087; p=0.768; n=14	rho=-0.125; p=0.669; n=14	rho=0.357; p=0.432; n=7	rho=-0.121; p=0.666; n=15	rho=-0.581; p=0.011; n=18	rho=0.260; p=0.298; n=18	rho=-0.025; p=0.927; n=16
**Serum total tau at T0**	rho=0.116; p=0.648; n=18	rho=0.043; p=0.864; n=18	rho=-0.276; p=0.268; n=18	rho=-0.264; p=0.432; n=11	rho=-0.243; p=0.402; n=14	rho=0.144; p=0.624; n=14	rho=0.018; p=0.951; n=14	rho=-0.004; p=0.988; n=14	rho=0.000; p=1.000; n=7	rho=-0.313; p=0.256; n=15	rho=0.465; p=0.052; n=18	rho=-0.012; p=0.963; n=18	rho=0.483; p=0.058; n=16
**CSF GFAP at T0**	rho=0.247; p=0.256; n=23	rho=0.247; p=0.256; n=23	rho=0.012; p=0.957; n=23	rho=0.161; p=0.552; n=16	rho=-0.412; p=0.079; n=19	rho=-0.193; p=0.429; n=19	rho=0.142; p=0.561; n=19	rho=-0.003; p=0.991; n=19	rho=0.336; p=0.312; n=11	rho=-0.201; p=0.423; n=18	rho=-0.107; p=0.627; n=23	rho=0.101; p=0.645; n=23	rho=-0.396; p=0.104; n=18
**CSF NfL at T0**	rho=-0.088; p=0.690; n=23	rho=-0.143; p=0.514; n=23	rho=-0.678; p<0.001; n=23	rho=0.006; p=0.983; n=16	rho=-0.137; p=0.575; n=19	rho=-0.155; p=0.526; n=19	rho=0.106; p=0.665; n=19	rho=-0.295; p=0.221; n=19	rho=-0.500; p=0.117; n=11	rho=-0.781; p<0.001; n=18	rho=0.102; p=0.645; n=23	rho=0.721; p<0.001; n=23	rho=0.129; p=0.610; n=18
**CSF UCHL-1 at T0**	rho=0.243; p=0.264; n=23	rho=0.231; p=0.288; n=23	rho=-0.354; p=0.098; n=23	rho=0.273; p=0.306; n=16	rho=-0.334; p=0.163; n=19	rho=-0.415; p=0.077; n=19	rho=0.259; p=0.285; n=19	rho=-0.061; p=0.803; n=19	rho=-0.136; p=0.689; n=11	rho=-0.399; p=0.101; n=18	rho=0.108; p=0.624; n=23	rho=0.358; p=0.094; n=23	rho=-0.246; p=0.325; n=18
**CSF total tau at T0**	rho=0.290; p=0.180; n=23	rho=0.324; p=0.131; n=23	rho=0.102; p=0.644; n=23	rho=0.217; p=0.420; n=16	rho=-0.364; p=0.126; n=19	rho=-0.360; p=0.130; n=19	rho=0.296; p=0.219; n=19	rho=0.069; p=0.778; n=19	rho=0.155; p=0.650; n=11	rho=-0.168; p=0.505; n=18	rho=-0.151; p=0.491; n=23	rho=0.045; p=0.837; n=23	rho=-0.464; p=0.053; n=18

Table S2. Baseline correlations between biomarkers.

Biomarker	Serum GFAP at T0	Serum NfL at T0	Serum UCHL-1 at T0	Serum Tau at T0	CSF GFAP at T0	CSF NfL at T0	CSF UCHL-1 at T0	CSF Tau at T0
**Serum GFAP at T0**	NA	rho=0.187; p=0.458; n=18	rho=0.571; p=0.013; n=18	rho=-0.036; p=0.887; n=18	rho=0.757; p<0.001; n=17	rho=0.025; p=0.926; n=17	rho=0.311; p=0.224; n=17	rho=0.385; p=0.127; n=17
**Serum NfL at T0**	rho=0.187; p=0.458; n=18	NA	rho=0.422; p=0.081; n=18	rho=-0.040; p=0.874; n=18	rho=0.047; p=0.859; n=17	rho=0.814; p<0.001; n=17	rho=0.422; p=0.092; n=17	rho=-0.091; p=0.729; n=17
**Serum UCHL-1 at T0**	rho=0.571; p=0.013; n=18	rho=0.422; p=0.081; n=18	NA	rho=-0.480; p=0.044; n=18	rho=0.596; p=0.012; n=17	rho=0.275; p=0.286; n=17	rho=0.483; p=0.050; n=17	rho=0.539; p=0.026; n=17
**Serum Tau at T0**	rho=-0.036; p=0.887; n=18	rho=-0.040; p=0.874; n=18	rho=-0.480; p=0.044; n=18	NA	rho=-0.227; p=0.381; n=17	rho=0.065; p=0.804; n=17	rho=-0.238; p=0.358; n=17	rho=-0.478; p=0.052; n=17
**CSF GFAP at T0**	rho=0.757; p<0.001; n=17	rho=0.047; p=0.859; n=17	rho=0.596; p=0.012; n=17	rho=-0.227; p=0.381; n=17	NA	rho=0.102; p=0.644; n=23	rho=0.645; p<0.001; n=23	rho=0.712; p<0.001; n=23
**CSF NfL at T0**	rho=0.025; p=0.926; n=17	rho=0.814; p<0.001; n=17	rho=0.275; p=0.286; n=17	rho=0.065; p=0.804; n=17	rho=0.102; p=0.644; n=23	NA	rho=0.560; p=0.005; n=23	rho=0.191; p=0.383; n=23
**CSF UCHL-1 at T0**	rho=0.311; p=0.224; n=17	rho=0.422; p=0.092; n=17	rho=0.483; p=0.050; n=17	rho=-0.238; p=0.358; n=17	rho=0.645; p<0.001; n=23	rho=0.560; p=0.005; n=23	NA	rho=0.773; p<0.001; n=23
**CSF Tau at T0**	rho=0.385; p=0.127; n=17	rho=-0.091; p=0.729; n=17	rho=0.539; p=0.026; n=17	rho=-0.478; p=0.052; n=17	rho=0.712; p<0.001; n=23	rho=0.191; p=0.383; n=23	rho=0.773; p<0.001; n=23	NA

Table S3. Responder subgroup analysis in the bottom tertile of CSF NfL ratio at Month 3 (T3/T0).

	Baseline (T0)	Month 3 (T3)	Month 6 (T6)	Last Administration
		**Serum**		
**Serum UCHL1**	33.47 [23.64] (n=7) GMRΔ +0.0% (n_pair=NA)	35.37 [21.90] (n=7) GMRΔ +7.9% (n_pair=7)	44.47 [9.95] (n=6) GMRΔ +19.3% (n_pair=6)	47.43 [12.09] (n=7) GMRΔ +38.5% (n_pair=7)
**Serum NfL**	34.92 [48.30] (n=7) GMRΔ +0.0% (n_pair=NA)	21.54 [9.19] (n=7) GMRΔ -24.1% (n_pair=7)	15.00 [6.19] (n=6) GMRΔ -33.3% (n_pair=6)	19.65 [17.13] (n=7) GMRΔ -35.9% (n_pair=7)
**Serum GFAP**	122.08 [164.76] (n=7) GMRΔ +0.0% (n_pair=NA)	173.10 [203.84] (n=7) GMRΔ +38.7% (n_pair=7)	129.49 [230.74] (n=6) GMRΔ +41.4% (n_pair=6)	150.33 [325.83] (n=7) GMRΔ +67.7% (n_pair=7)
**Serum Total Tau**	0.73 [0.62] (n=7) GMRΔ +0.0% (n_pair=NA)	1.50 [1.51] (n=7) GMRΔ +13.5% (n_pair=7)	0.65 [0.78] (n=6) GMRΔ -2.6% (n_pair=6)	0.61 [1.18] (n=7) GMRΔ +10.8% (n_pair=7)
		**CSF**		
**CSF UCHL1**	2068.11 [1799.02] (n=8) GMRΔ +0.0% (n_pair=NA)	1961.24 [1193.69] (n=8) GMRΔ -13.1% (n_pair=8)	2939.49 [2315.44] (n=8) GMRΔ +9.2% (n_pair=8)	3706.05 [3888.62] (n=8) GMRΔ +52.4% (n_pair=8)
**CSF NfL**	3896.15 [3868.80] (n=8) GMRΔ +0.0% (n_pair=NA)	1989.52 [2159.51] (n=8) GMRΔ -51.3% (n_pair=8)	2351.54 [2726.10] (n=8) GMRΔ -45.5% (n_pair=8)	1570.43 [2518.96] (n=8) GMRΔ -52.6% (n_pair=8)
**CSF GFAP**	7486.52 [13000.36] (n=8) GMRΔ +0.0% (n_pair=NA)	9008.15 [12070.54] (n=8) GMRΔ +7.8% (n_pair=8)	14101.69 [19489.39] (n=8) GMRΔ +50.8% (n_pair=8)	17862.17 [16085.00] (n=8) GMRΔ +83.5% (n_pair=8)
**CSF Total Tau**	63.52 [25.65] (n=8) GMRΔ +0.0% (n_pair=NA)	60.86 [32.20] (n=8) GMRΔ -13.3% (n_pair=8)	71.53 [64.95] (n=8) GMRΔ -6.7% (n_pair=8)	88.00 [44.19] (n=8) GMRΔ +33.1% (n_pair=8)

Table S4. Univariable and multivariable linear regression models of long-term DPR ratio (LA/T0) according to month-6 serum biomarker changes.

Linear regression models using raw within-patient biomarker ratios (T6/T0)

Model	Variable	β (95% CI)	p-value	n	R^2	Adj. R^2
		**Serum UCHL-1 ratio (Month 6 / baseline)**				
**Univariate model**	Serum UCHL-1 ratio (T6/T0)	0.318 (0.113 to 0.524)	0.006	13	0.513	0.469
		**Serum NfL ratio (Month 6 / baseline)**				
**Univariate model**	Serum NfL ratio (T6/T0)	0.449 (0.032 to 0.866)	0.037	13	0.338	0.278
		**Serum UCHL-1 ratio + Serum NfL ratio (Month 6 / baseline)**				
**Multivariable model**	Serum UCHL-1 ratio (T6/T0)	0.386 (-0.054 to 0.827)	0.079	13	0.521	0.425
**Multivariable model**	Serum NfL ratio (T6/T0)	-0.136 (-0.903 to 0.631)	0.701	13	0.521	0.425

Outcome: DPR ratio (LA/T0). Predictors are within-patient biomarker ratios at Month 6 relative to baseline.

Serum UCHL-1 = SUCH; Serum NfL = SNFL.

Table S5. Baseline and longitudinal NfL/GFAP ratio in serum and CSF during tofersen treatment.

Biomarker	Baseline (T0)	Month 3 (T3)	Month 6 (T6)	Last administration (LA)
**Serum NfL/GFAP ratio**	0.27 [0.40] (n=18)	0.17 [0.32] (n=17); GMRΔ -33.3% (n_pair=17)	0.13 [0.09] (n=13); GMRΔ -47.2% (n_pair=13)	0.10 [0.14] (n=18); GMRΔ -53.0% (n_pair=18)
**CSF NfL/GFAP ratio**	0.37 [0.60] (n=23)	0.19 [0.34] (n=22); GMRΔ -43.6% (n_pair=22)	0.17 [0.24] (n=22); GMRΔ -57.0% (n_pair=22)	0.11 [0.11] (n=23); GMRΔ -66.0% (n_pair=23)

Table S6. Timepoint-specific correlations between biomarker changes and CSF total protein changes during tofersen treatment. Spearman rank correlations between within-participant biomarker ratios and CSF total protein ratios were calculated at each post-baseline timepoint. For each biomarker, changes were expressed as ratios relative to baseline, corresponding to T3/T0, T6/T0, and LA/T0. The table reports the number of paired observations available for each comparison, Spearman's rho, and Holm-adjusted p values.

Biomarker	Comparison	n	rho	p value
**Serum NfL**	T3/T0	6	0.42857142857142900	1
**Serum NfL**	T6/T0	3	NA	NA
**Serum NfL**	LA/T0	7	-0.3571428571428570	1
**Serum GFAP**	T3/T0	6	0.6571428571428570	1
**Serum GFAP**	T6/T0	3	NA	NA
**Serum GFAP**	LA/T0	7	-0.8214285714285710	0.4455273585681400
**Serum UCHL-1**	T3/T0	6	0.028571428571428600	1
**Serum UCHL-1**	T6/T0	3	NA	NA
**Serum UCHL-1**	LA/T0	7	0.46428571428571400	1
**Serum total tau**	T3/T0	6	-0.2571428571428570	1
**Serum total tau**	T6/T0	3	NA	NA
**Serum total tau**	LA/T0	7	0.21428571428571400	1
**CSF NfL**	T3/T0	7	-0.21428571428571400	1
**CSF NfL**	T6/T0	7	0.46428571428571400	1
**CSF NfL**	LA/T0	8	0.16666666666666700	1
**CSF GFAP**	T3/T0	7	-0.607142857142857	1
**CSF GFAP**	T6/T0	7	0.9642857142857140	0.009082983383883650
**CSF GFAP**	LA/T0	8	0.42857142857142900	1
**CSF UCHL-1**	T3/T0	7	0.17857142857142900	1
**CSF UCHL-1**	T6/T0	7	0.5357142857142860	1
**CSF UCHL-1**	LA/T0	8	0.28571428571428600	1
**CSF total tau**	T3/T0	7	-0.14285714285714300	1
**CSF total tau**	T6/T0	7	0.7142857142857140	1
**CSF total tau**	LA/T0	8	-0.380952380952381	1

Figure S1. Sankey plot showing transitions across DPR-based progression groups from pre-tofersen to last administration.

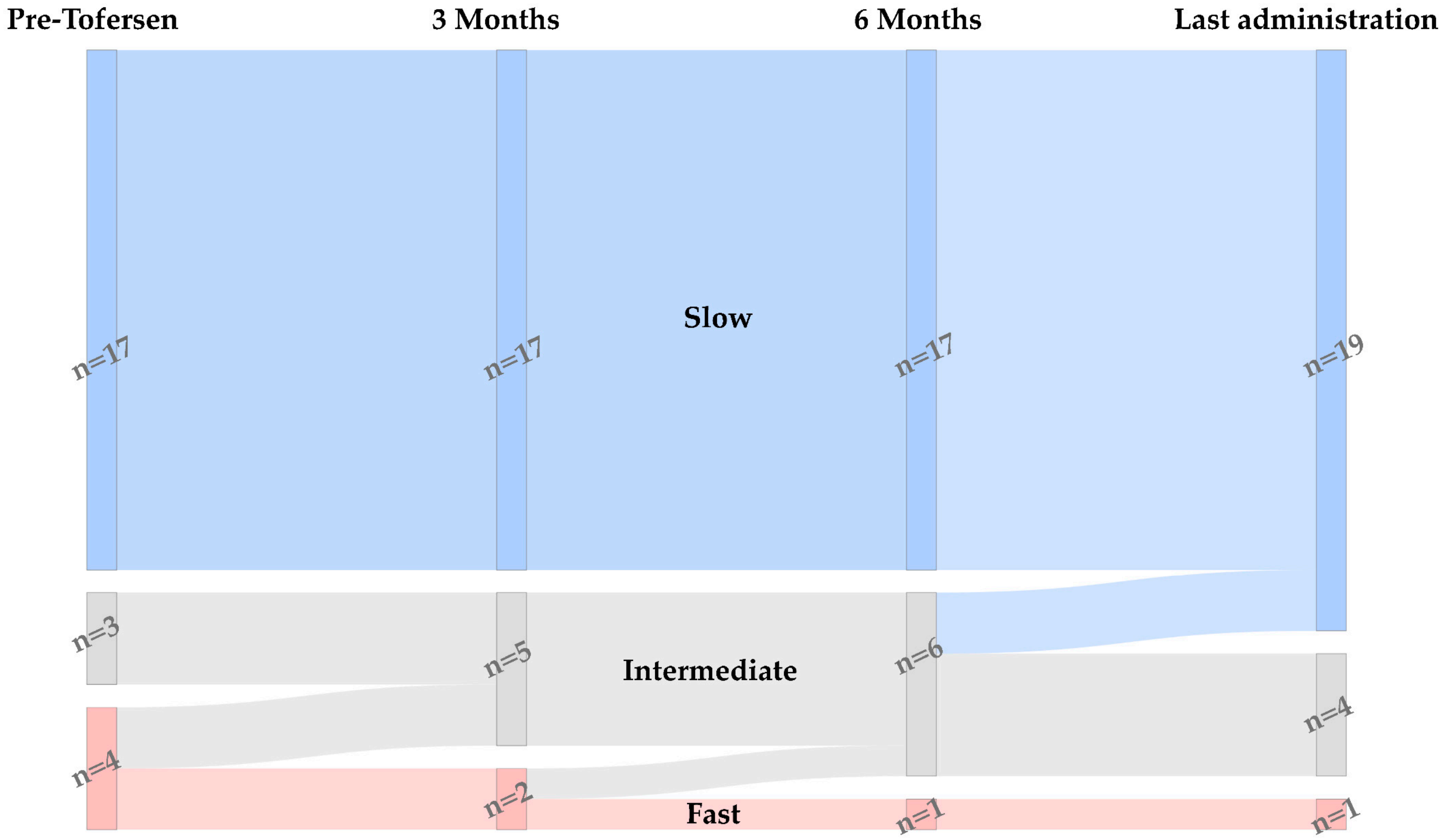

Figure S2. Longitudinal geometric mean ratio (GMR) profiles of serum NfL and serum UCHL1 in patients with greater versus lesser DPR reduction during tofersen treatment. Significance is indicated as * = p < 0.05, ** = p < 0.01.

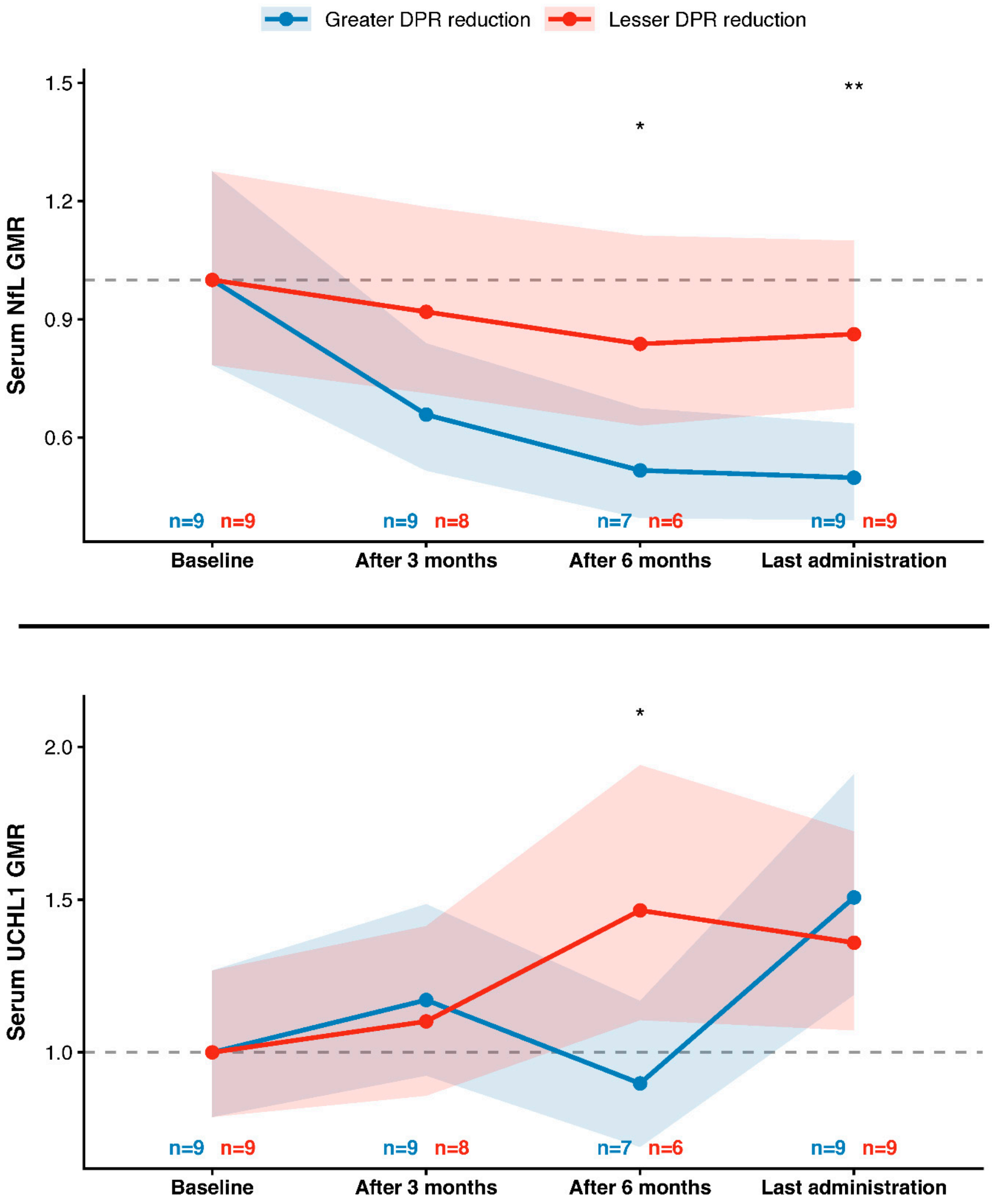

Figure S3. CSF and Serum biomarker trajectories in the bottom tertile of CSF NfL ratio at Month 3 (T3/T0). Significance is indicated as * = p < 0.05, ** = p < 0.01, ns = not significant.

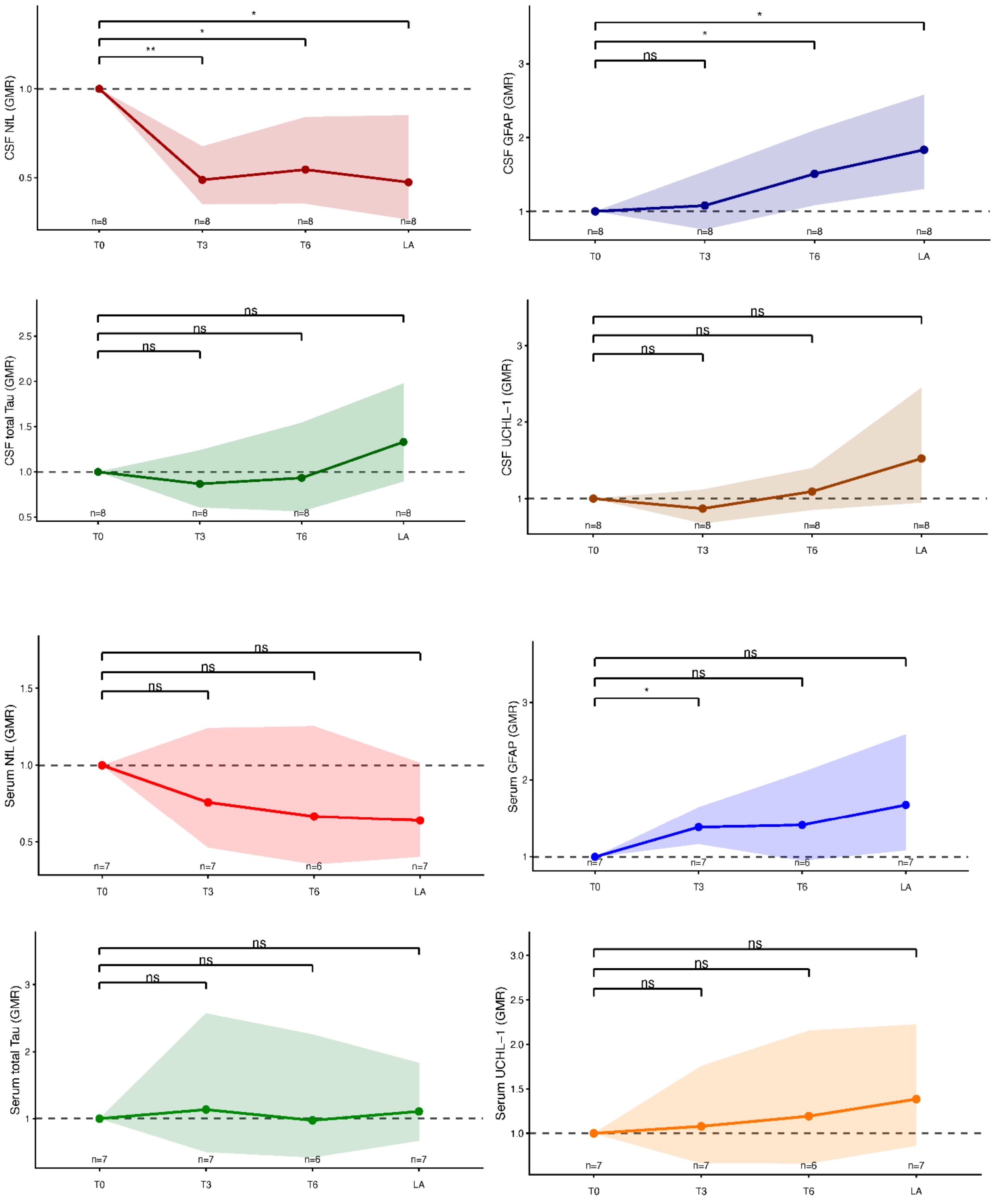

Figure S4. Serum and CSF NfL/GFAP ratio trajectories during Tofersen Treatment. Significance is indicated as *** = $p < 0.001$.

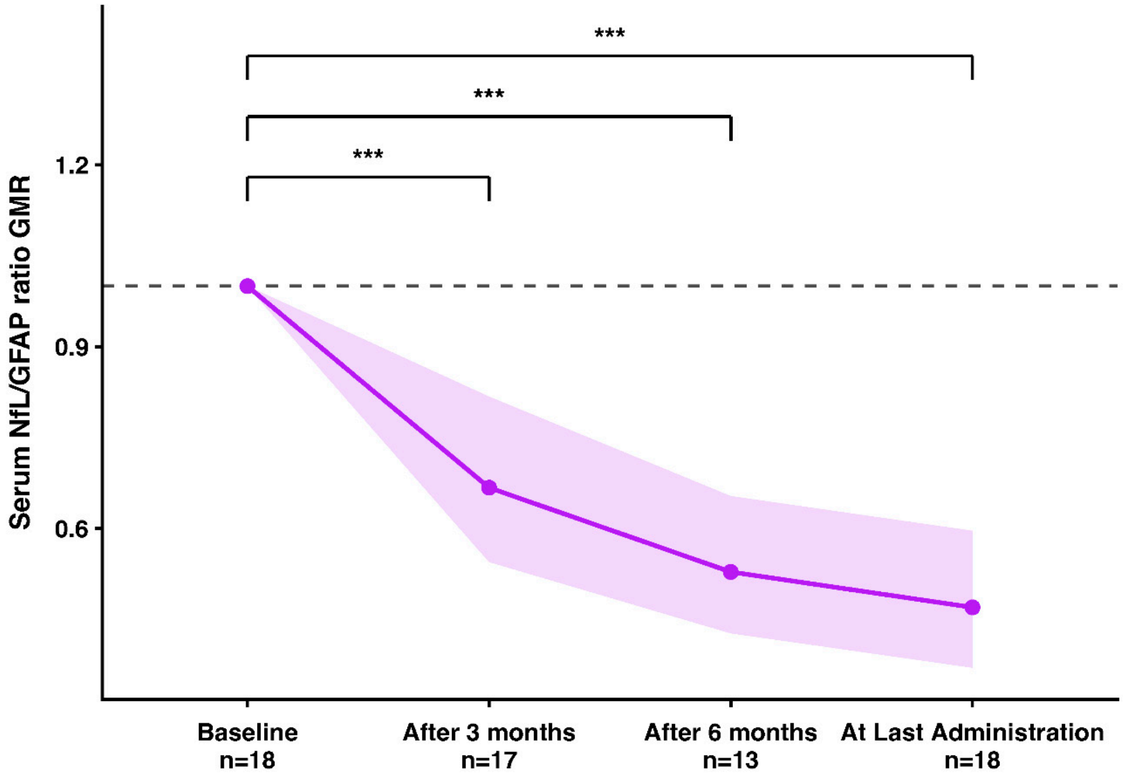

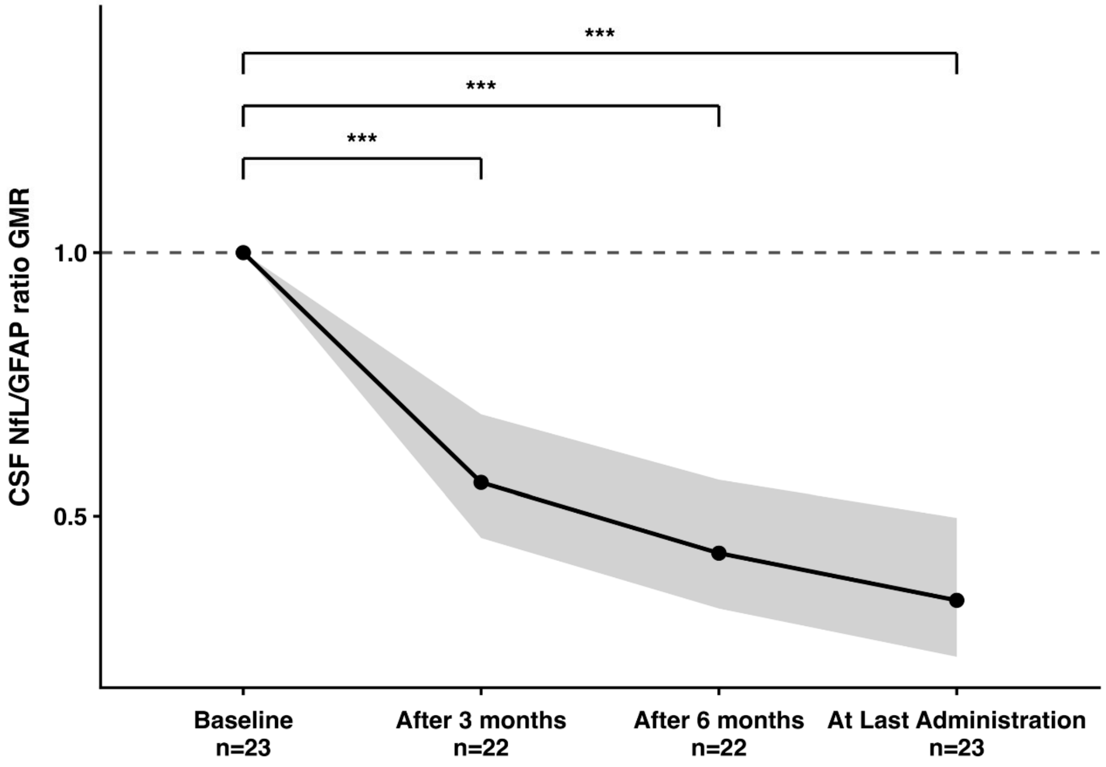

Figure S5. Longitudinal trajectories of CSF GFAP and CSF total proteins during tofersen treatment. Within-participant changes in CSF GFAP and CSF total proteins are shown as geometric mean ratios relative to baseline across follow-up timepoints. Thin lines represent individual patient trajectories, while thick lines indicate the cohort-level geometric mean ratio with 95% confidence intervals. Stars above timepoints denote timepoint-specific Spearman correlations between CSF GFAP and CSF total protein Tx/T0 ratios. Significance is indicated as *** = $p < 0.001$.

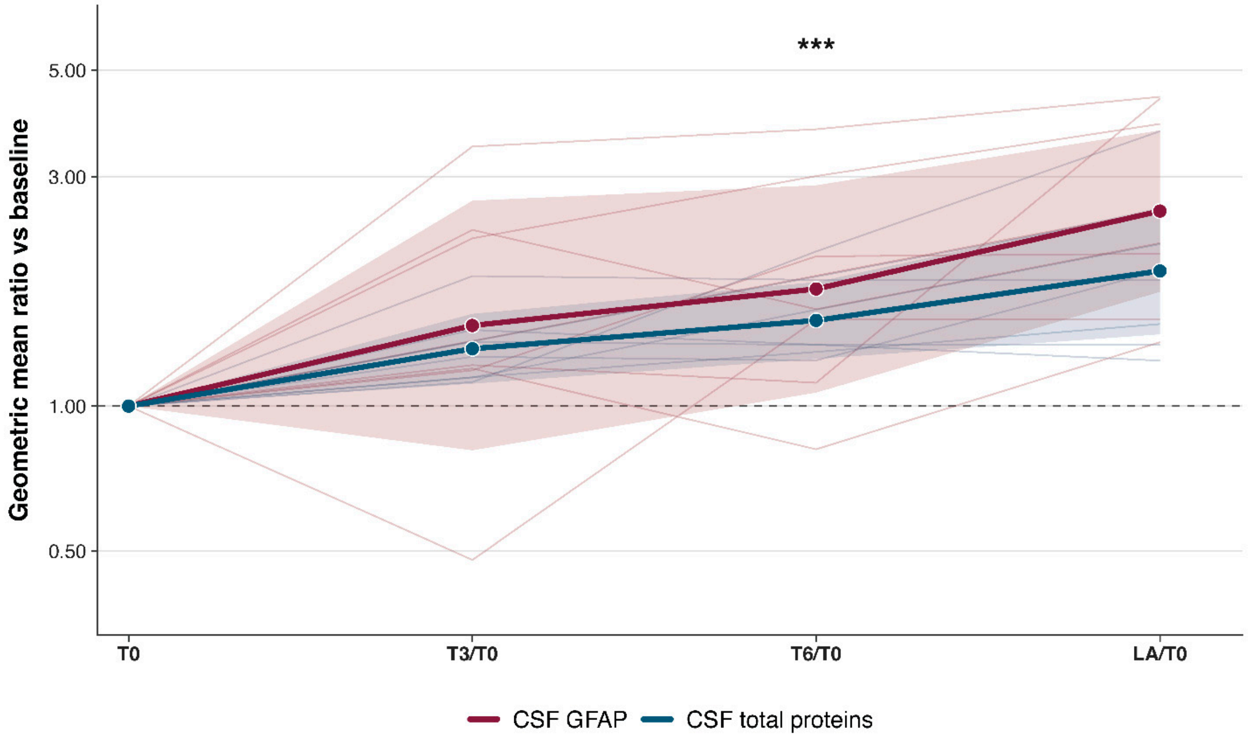

Supplement: Supplementary file 1 [file ijms-27-04208-s001.zip › ijms-4277406-supplementary.pdf]
